# Comprehensive Assessment of Nitrosamine Formation in Meat Products Using UHPLC-HRMS: Analytical Challenges and Potential Dietary Implications

**DOI:** 10.3390/molecules30204107

**Published:** 2025-10-16

**Authors:** Tiziana Nardin, Jakob Franceschini, Francesca Martinelli, Elena Franciosi, Roberto Larcher

**Affiliations:** 1 Centro Trasferimento Tecnologico, Fondazione E. Mach, Via E. Mach 1, 38098 San Michele all’Adige (TN), Italy; tiziana.nardin@fmach.it (T.N.); jakob.franceschini@hotmail.it (J.F.); francesca.martinelli@fmach.it (F.M.); 2 Centro Ricerca e Innovazione, Fondazione E. Mach, Via E. Mach 1, 38098 San Michele all’Adige (TN), Italy; elena.franciosi@fmach.it

**Keywords:** nitrosamines, UHPLC-HRMS, processed meat

## Abstract

Nitrosamines (NAs) pose a risk due to their carcinogenic properties, especially in processed and cured meats where nitrites and nitrates are widely used. The objective of this study was to develop an integrated Ultra-High-Performance Liquid Chromatography–High-Resolution Mass Spectrometry (UHPLC–HRMS) workflow for detecting both volatile (VNAs) and non-volatile (NVNAs) nitrosamines in meat matrices. Comparison of two ionization techniques showed that heated electrospray ionization (HESI) and atmospheric pressure chemical ionization (APCI) provided complementary coverage and sensitivity. Extraction and cleanup were optimized for meat, although recovery rates remained variable, underscoring the analytical complexity. The method was applied to raw, cooked, cured, and grilled meats, as well as to in vitro gastric digestion and co-digestion with spinach. Results revealed that some NAs were present even in untreated raw meat (≈3.0 µg/kg, N-nitrosodi-n-butylamine), while the addition of nitrites and nitrates significantly increased their levels (more than 10 µg/kg, N-nitrosodiethylamine, N-nitrosodimethylamine, N-nitrosodi-n-butylamine). Gastric digestion was the most critical condition, further promoting nitrosamine formation, particularly for N-nitrosodiethylamine, N-nitrosodi-n-butylamine, and N-nitrosopiperidine. Ascorbate exhibited a dual role, acting as an inhibitor at low nitrite concentrations but becoming pro-oxidant at high levels (300 mg/kg). Cooking alone had limited impact, whereas cooking combined with digestion yielded the highest and most consistent nitrosamine concentrations. The inclusion of spinach during digestion modestly altered nitrosamine levels, reflecting both its nitrate content and polyphenolic profile. Nonparametric ANOVA (aligned rank transform) confirmed that preservative treatment, rather than processing or interaction effects, was the main driver of variability (total nitrosamines: H = 24.15, *p* = 2.33 × 10^−5^), with the combination of preservative ascorbate plus nitrite producing significantly higher levels than other treatments (q = 0.000656). N-nitrosodimethylamine consistently emerged as the most relevant marker for dietary exposure, in agreement with EFSA guidance. Overall, this study underscores both the analytical and biochemical complexity of nitrosamine detection and formation in meat products, while highlighting the importance of preservative formulation and the potential role of dietary antioxidants in mitigating exposure.

## 1. Introduction

Nitrosamines (NAs) are a class of organic compounds characterized by the general formula R_1_R_2_N–N=O [1]. Structurally, they can be described as resonance hybrids, with one canonical form represented as R_1_R_2_N–N=O ↔ R_1_R_2_N^+^=N–O^−^. These compounds are typically formed through the nitrosation of secondary amines, a reaction facilitated by nitrosating agents. Due to their ubiquitous presence in a wide range of consumer products, including food, beverages, and pharmaceuticals, NAs have come under increasing regulatory scrutiny, driven by growing concerns over their potential health risks, particularly their carcinogenicity [2]. The International Agency for Research on Cancer (IARC) has classified 24 NAs into different carcinogenic risk groups, with N-nitrosonornicotine (NNN) and 4-(N-nitrosomethylamino)-1-(3-pyridyl)-1-butanone (NNK) categorized as Group 1, carcinogenic to humans [3].

One of the sectors of greatest concern regarding NA formation is the meat industry, particularly in cured meats such as sausages, salami, and smoked products. In these products, nitrites and nitrates are commonly used as preservatives to enhance color and inhibit bacterial growth [4]. Many countries regulate nitrite use to minimize NA formation. In the EU, Commission Regulation (EU) 2023/2108 amends Annex II to Regulation (EC) No 1333/2008 and lowers permitted nitrite levels. From 9 October 2025, the maximum added amount is 80 mg/kg for meat products in general and 55 mg/kg for sterilized meat products (≈120 mg/kg and 82 mg/kg when expressed as sodium nitrite). Furthermore, cooking methods involving high temperatures, such as grilling or frying, can significantly increase NA levels in these products [5]. The stomach also plays a key role in nitrosation: NA precursors (secondary amines and nitrite) introduced with food and saliva are temporarily retained and mixed in an acidic environment that favors nitrosation [6].

Most existing research on NA formation and occurrence focuses primarily on volatile NAs (VNAs), with non-volatile NAs (NVNAs) being less commonly investigated. While many VNAs are confirmed carcinogens and most of the others are presumed to be carcinogenic, only a small number of NVNAs are considered likely to pose such risks [7]. However, emerging toxicological studies suggest that certain NVNAs, including N-nitroso-L-proline (NPRO) and N-nitrososarcosine (NSAR), may also present genotoxic or carcinogenic potential, and some of them are currently under evaluation by EFSA and IARC [8]. Despite this, systematic data on NVNA occurrence remain limited, making their toxicological relevance underexplored. It still remains challenging to find a study that provides an extensive evaluation of many volatile NAs, most likely due to the limited availability and high cost of the standards. Usually, the most investigated are NDMA, NPYR, NPIP, and NDEA.

Controlling the formation of NAs presents significant challenges, primarily due to the absence of standardized analytical methods [9] and the poor availability and intrinsic instability of these compounds. These factors collectively hinder the development of robust strategies to control NA formation in food products. Traditional detection methods have mainly relied on gas chromatography–mass spectrometry (GC-MS) or targeted LC-MS/MS, which are highly sensitive but generally limited to VNAs, overlooking many NVNAs [10,11,12]. In contrast, ultra-high-performance liquid chromatography coupled with high-resolution mass spectrometry (UHPLC–HRMS) offers broader compound coverage, enabling the simultaneous detection of both VNAs and NVNAs [13]. Moreover, the combination of heated electrospray ionization (HESI) and atmospheric pressure chemical ionization (APCI) provides complementary strengths: HESI enhances sensitivity for polar NVNAs, whereas APCI is more effective for VNAs. This dual-source strategy represents a key methodological advantage. Moreover, the ability to work in high resolution allows for greater selectivity with increased assurance of avoiding false positives.

In this study, we first developed an efficient analytical method for NA screening using UHPLC–HRMS, specifically a hybrid quadrupole–Orbitrap mass spectrometer (Q-Orbitrap). A key challenge was selecting the most suitable ionization source (HESI vs. APCI) to achieve low detection limits for both VNAs and NVNAs. We also developed a dedicated extraction–concentration–cleanup protocol tailored to meat samples, one of the key matrices under investigation. The method was then applied to screen NAs across fresh, processed, and cured meats, and to monitor their formation during storage, cooking, and digestion. This comprehensive approach was designed to improve our understanding of NA formation in food and to test the hypothesis that both volatile and non-volatile nitrosamines can be effectively profiled under realistic processing and digestion conditions, thereby supporting risk assessment and regulatory monitoring.

## 2. Results and Discussion

### 2.1. Method Validation

#### 2.1.1. Sample Extraction

Several extraction protocols were tested to optimize recovery efficiency. Initial trials with ACN/formic acid [7] and a modified ACN-based method [14], yielded poor recoveries (<15%). Although dichloromethane (DCM) showed better affinity, its high solvent demand made it less practical. The final protocol, adapted from Raoul et al. [15] offered the best compromise between recovery and solvent use and was therefore selected for subsequent analyses.

#### 2.1.2. Online SPE Concentration

The decision to implement an online solid phase extraction (SPE) system was based on the fact that the literature reports very low concentrations of NAs [16,17]. To address this issue, we opted for a concentration of the extract (paragraph 2.4), followed by an additional pre-concentration and matrix cleanup using online SPE. To identify the most suitable SPE cartridge, the following were tested: a Biphenyl SPE (Kinetex 5 µm, 4.0 × 20 mm; Phenomenex, Torrance, CA, USA), a C18 SPE (Strata 20 µm, 2.0 × 20 mm, Phenomenex), a HILIC SPE (Luna 5 µm, 2.0 × 10 mm, Phenomenex), a DVB SPE (SolEx HRP 2.1 × 20 mm, Thermo Fisher Scientific) and a PEP SPE (divinylbenzene functionalized with urea groups) (HyperSep Retain PEP Polymeric Material, 57 µm, 1.5 × 15 mm, Thermo Fisher Scientific, Waltham, MA, USA).

A mixture of standards was injected to evaluate SPE performance. Retention times in Table 1 were recorded during reverse-flush under the analytical gradient and therefore reflect the apparent elution window from the SPE cartridge. Most cartridges (Biphenyl, C18, HILIC) showed very early elution (0.15–0.20 min), indicating poor retention. In contrast, DVB and PEP delayed the first peak to about 0.4 min, confirming higher affinity for NAs. PEP displayed a narrower retention window (0.4–1.8 min), whereas DVB offered a broader range (0.4–2.2 min) and thus greater overall capacity. Based on these results, DVB was selected for subsequent analyses, with a matrix wash time of 0.3 min.

#### 2.1.3. Chromatographic Separation

Considering the number of NAs tested and their differing reactivities, several chromatographic columns were evaluated using a gradient of H_2_O with 0.4% acetic acid and acetonitrile [18]. The Poroshell 120 EC-C18 (2.7 µm, 3.0 × 100 mm; Agilent Technologies, Santa Clara, CA, USA) provided symmetric peaks but most analytes eluted too early (~1.25 min), reducing sensitivity. The Accucore Polar Premium (2.6 µm, 3.0 × 150 mm, Thermo Fisher Scientific), with amide groups, improved retention and efficiency but showed peak splitting. The Hypersil Gold Q-A (1.9 µm, 2.1 × 100 mm, Thermo Fisher Scientific), with a highly porous matrix, further reduced retention times and efficiency, making it less suitable. The Acclaim Vanquish PA2 column (2.2 µm, 2.1 × 150 mm, Thermo Fisher Scientific), designed for complex matrices, showed the best overall performance, with balanced retention, superior peak separation, and high theoretical plate counts, although some peak splitting persisted. Comparative metrics (RT, K′, asymmetry, plates) are reported in Table 2. Overall, the PA2 column provided the most consistent and reproducible efficiency across the NA panel, justifying its selection as the optimal stationary phase. The PA2 column was therefore preferred over APP not only for higher peak areas, but also for its more uniform selectivity toward polar NVNAs, improved peak shape and symmetry, and greater robustness across the entire analyte panel.

#### 2.1.4. APCI vs. HESI

The pre-concentration and matrix-cleanup technique using SPE (DVB column) was combined with chromatographic separation on the Acclaim Vanquish PA2 column to achieve the most sensitive and effective separation of the investigated NAs. Identification of NAs was based on their corresponding protonated [M+H]^+^ or deprotonated [M−H]^−^ molecular ions (within a ± 5 ppm mass error) in full-scan MS-data—dependent MS/MS (dd-MS/MS) mode. Retention time (RT; tolerance ±0.5 min), isotopic pattern, and fragmentation spectra acquired served as confirmatory tools. Both HESI and APCI sources were tested.

APCI and HESI were run as separate injections. For most NAs, APCI yielded higher response than HESI (Table 3); exceptions included NDPhA, which was undetectable by APCI, plausibly due to in-source fragmentation [19], or poor protonation efficiency, and NNN/NDBA, which ionized better in HESI.

The recovery data obtained for the tested NAs reveal distinct differences in the performance of APCI and HESI sources. Several compounds, such as NDMA, NPRO, NDPA, and NPIP, exhibited consistently low recoveries across both ionization modes, with values below 40%. In particular, NDMA was completely undetected in HESI mode, suggesting possible degradation or ionization inefficiency under these conditions. Since our aim was not to focus on a restricted set of nitrosamines but to cover the widest possible spectrum, the current method represents the best compromise available. Intermediate recoveries, ranging between 40% and 70%, were observed for compounds including NSAR, NMEA, and NPYR, indicating moderate extraction efficiency and acceptable ionization performance, although further optimization may be warranted. Notably, several NAs displayed significantly higher recovery using APCI. For example, NNK showed a recovery of 85% in APCI, compared to only 15% in HESI. Similarly, NMTCA presented an anomalously high recovery of 155% with APCI, possibly indicating a ion enhancement. These results support the greater sensitivity of APCI for certain NAs, particularly nonpolar or moderately polar species. In contrast, NDPhA was not detected under APCI but was quantifiable using HESI (35% recovery), suggesting that APCI may induce in-source fragmentation for thermolabile or structurally sensitive compounds. Additionally, compounds such as NNN and NDBA also showed enhanced peak areas and better ionization with HESI. These findings indicate that while APCI generally offers higher sensitivity for many NAs, HESI is better suited for more fragile or polar analytes. A dual-ionization strategy utilizing both APCI and HESI may be necessary to ensure comprehensive coverage and accurate quantification of all NAs of interest. The results were corrected with matrix recovery and uncertainties were estimated for quantified NA as 19% for NDEA, 29% for NDBA, 36% for NDPA, 25% for NPIP, 45% NPYR and 16% for NMOR.

**Figure 1:**
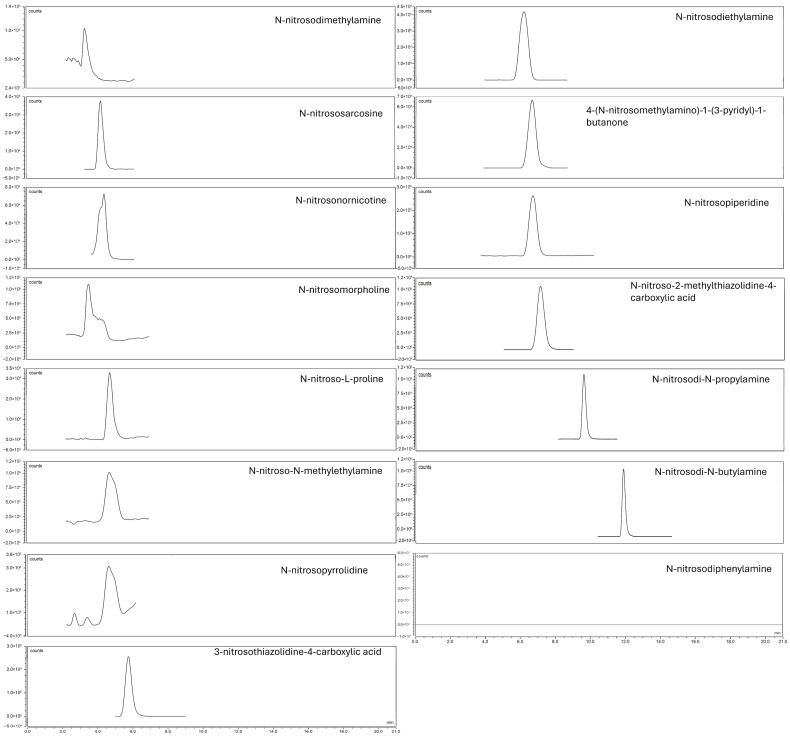
APCI chromatogram of 5 µg/L standard solution.

**Figure 2:**
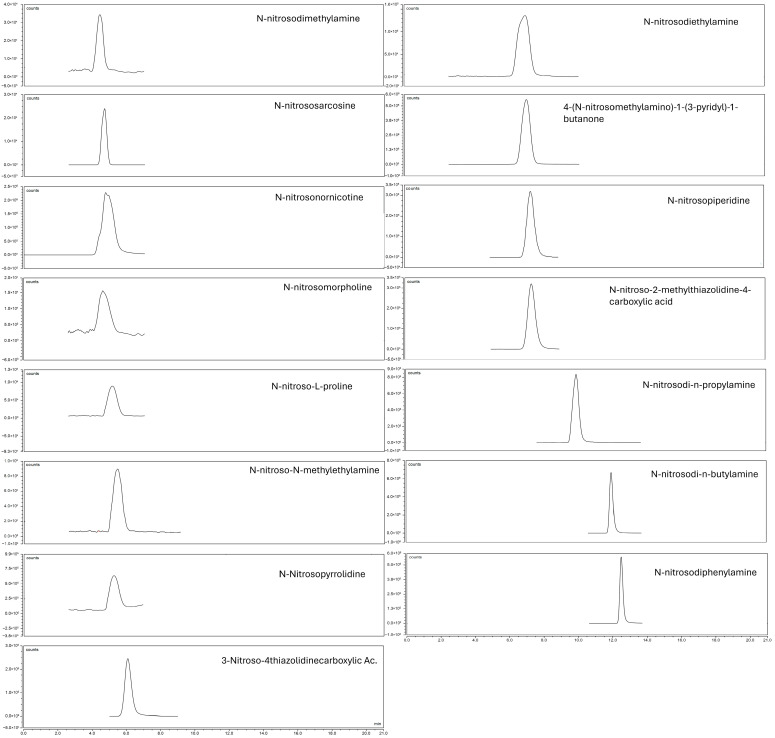
HESI chromatogram of 5 µg/L standard solution.

Figure 1 and Figure 2 show the chromatograms of 5 µg/L analytical standards obtained with APCI and HESI, respectively.

### 2.2. NA Formation

The evaluation of NA levels in various meat treatments, raw, digested, cooked, cooked and digested, and cooked and digested with spinach, reveals distinct trends in their formation, influenced by the presence of additives (ASC, nitrites/nitrates) and processing stages (heating, digestion) (Table 4). Among the various NAs evaluated, NDPhA, NSAR, NPRO, NMEA, NNN, NNK, NTCA, and NMTCA were never quantified at levels above the limit of detection (LOD). NDMA, NDEA, NDPA, NPIP, and NDBA were quantified using an APCI source. All these molecules were confirmed by the presence of their characteristic fragmentation peaks, except for NDMA and NDEA, for which the fragmentation signal was too weak to provide reliable confirmation. NPYR and NMOR were quantified using an HESI source.

#### 2.2.1. NA Formation in Raw Meat

Unexpectedly, measurable levels of certain NAs were detected even in freshly cut, untreated raw meat, indicating that endogenous precursors and naturally occurring nitrosating agents may contribute to basal NA presence. This observation supports the hypothesis that endogenous amines and nitrosating agents possibly generated through microbial nitrate reduction during post-mortem storage can initiate low-level nitrosamine formation even under mild conditions [20]. NMOR was detected exclusively in the two ascorbate-treated samples, suggesting a possible interaction between ascorbate and endogenous precursors. The addition of nitrites and nitrates significantly increased NA formation in agreement with previous findings [16,17,21], with the ASC + NO_2_ combination producing markedly higher levels, particularly for NDBA (32 µg/kg) and NDMA (23 µg/kg). Interestingly, while ascorbate is generally known to inhibit nitrosamine formation by reducing nitrites [22], at higher NO_2_ concentrations (300 mg/kg, higher than allowed dose) it may actually promote their formation becoming a source of nitrosating radicals (NO• and NO_2_•) that can combine to generate NO^+^ or directly nitrosate amines [23]. Moreover, if traces of iron or copper, common in meat extracts, are present in the system, ascorbate reduces Fe^3+^ to Fe^2+^, which in turn catalyzes the oxidation of NO_2_^−^ to NO_2_•; this generates even more reactive species capable of nitrosating amines [22]. In samples treated with ASC alone, nitrosamine levels remained marginal, supporting the hypothesis of a dose-dependent pro-oxidant effect modulated by nitrite concentration.

#### 2.2.2. NA Formation in Digested Raw Meat

Simulated gastric digestion markedly increased nitrosamine formation, especially for NDEA and NDBA. NPIP reached about 50 µg/kg in ASC + NO_2_ samples, consistent with the high reactivity of cyclic secondary amines in acidic nitrosating environments. In fact, previous studies [20,24,25] have confirmed that the acidic gastric environment is optimal for nitrosation, particularly in the presence of nitrosating agents and reducing compounds like ascorbate. The ambiguous role of ascorbate is context-dependent: at low nitrite levels and in the absence of pro-oxidant cofactors, ascorbate efficiently inhibits nitrosation by scavenging nitrosating species; however, under gastric-like conditions with high nitrite and lipid/transition-metal presence, its behavior can shift toward pro-oxidant pathways, enhancing radical formation and thereby promoting N-nitroso compound generation. Refs. [26,27]. Some nitrosamines, such as NDEA, appeared only in digested samples and not in cooked meat, suggesting they are thermolabile or unstable under non-acidic conditions. This is consistent with Scanlan [28], who reported that many nitrosamines form primarily in simulated gastric environments and are sensitive to pH and temperature.

In this work, in vitro digestion was limited to the gastric phase, since acidic conditions are the most critical for nitrosation and nitrosamine formation. This choice is consistent with established evidence that nitrosation occurs most commonly under acidic conditions, whereas neutral to alkaline environments are generally less favorable for the generation of nitrosating species [29].

#### 2.2.3. NA Formation in Cooked Meat

Cooking without subsequent digestion did not lead to substantial NA formation, with cumulative concentrations rarely exceeding 30 µg/kg. Thermal energy can drive nitrosation at high temperatures, but in the absence of an acidic medium the equilibrium tends to favor nitrite decomposition to NO and NO_2_ gases rather than direct nitrosation.

Nevertheless, certain nitrosamines (NDBA, NDPA) still formed in nitrite-treated samples, potentially due to localized hot spots and microenvironments where moisture, pH, and amine concentration are optimal for reaction. Literature reports also suggest that lipid oxidation during heating can yield aldehydes that react with amines to form heterocyclic amines, which may then undergo nitrosation upon residual nitrite exposure [30]. The lower levels here compared to digestion conditions highlight that temperature alone is less influential than pH and nitrite availability.

#### 2.2.4. NA Formation in Cooked Digested Meat

**Figure 3:**
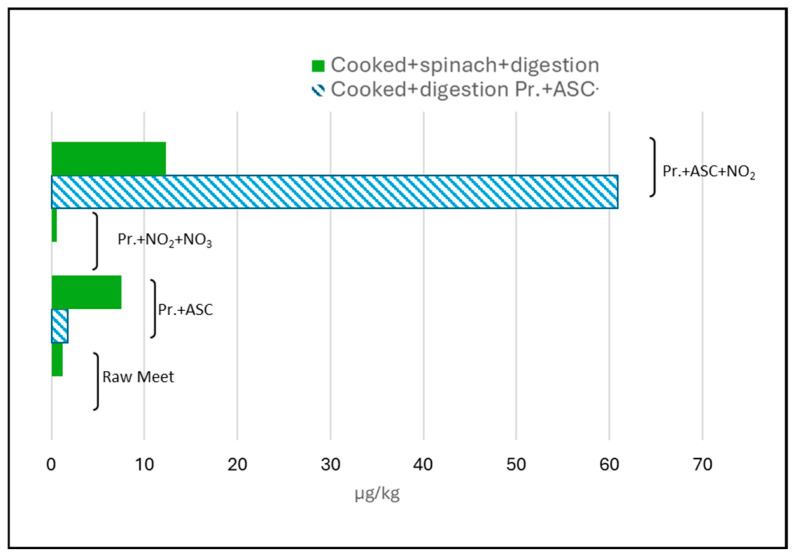
Comparison of nitrosamine sum concentrations in cooked and digested meat with and without spinach addition. Pr. = processed; ASC = ascorbic acid.

This group represents the most realistic experimental condition regarding human exposure and showed the highest reproducibility across replicates, suggesting a more stable kinetic profile of nitrosamine formation under physiologically simulated conditions. NPIP exceeded 40 µg/kg on average, and NDEA, NDBA, NDPA were frequent, particularly in ASC + NO_2_ treatments. The interplay of heat-induced precursor release and acid-promoted nitrosation likely explains this synergistic effect. The role of ascorbate is again ambiguous: while it may inhibit certain nitrosamines through its antioxidant effect, it can also catalyze the formation of reactive nitrosating intermediates in presence of nitrites [22]. The addition of spinach during digestion, as a source of vegetable nitrates, altered the NA profile: NDEA and NDMA increased, albeit remaining at low absolute concentrations (≤18 µg/kg in ASC + NO_2_; Figure 3). In particular, considering the total NA content, an increase of 1 µg/kg was observed in untreated meat, 6 µg/kg in meat treated only with ASC, and a decrease of 59 µg/kg in meat treated with both ASC and NO_2_. This aligns with findings by Grosse et al. [31] that vegetable nitrates alone are insufficient for NA formation unless nitrites or catalytic cofactors are present. Phenolic compounds in spinach may have a dual role: at low nitrosating stress, they scavenge reactive species, but at higher oxidative loads, they can undergo redox cycling, transiently enhancing radical-mediated nitrosation [32]. From a toxicological standpoint, even these low concentrations are relevant given the high carcinogenic potency of certain NAs (e.g., NDMA). Chronic exposure assessments must account for cumulative intake from multiple food sources and possible synergistic effects with endogenous formation in the gastrointestinal tract.

### 2.3. Statistical and Dietary Considerations

The nonparametric ANOVA (aligned rank transform, ART) and related post hoc tests indicated that total nitrosamines (SUM) were not influenced by processing (Kruskal–Wallis H = 3.50, *p* = 0.4775; ε^2^ ≈ 0), suggesting that raw, cooked, or digested matrices per se do not determine nitrosamine variability. By contrast, preservative treatment emerged as the major determinant (H = 24.15, *p* = 2.33 × 10^−5^; ε^2^ ≈ 0.59, large). Post hoc Mann–Whitney tests with FDR-BH correction confirmed that Pr. + ASC + NO_2_ produced significantly higher SUM levels compared with untreated meat, Pr. + ASC alone, or Pr. + NO_2_ + NO_3_ (q = 0.000656 for all three contrasts). At the level of individual compounds, only NDEA showed some effect of processing (H = 14.07, *p* = 0.0072), but this association was borderline after correction (q = 0.0505), and all other nitrosamines were unaffected. Across treatments, however, several analytes exhibited strong increases: NDBA (H = 24.47, *p* = 2.22 × 10^−5^; q = 5.06 × 10^−5^), NDPA (H = 24.49, *p* = 2.18 × 10^−5^; q = 2.25 × 10^−5^), NPIP (H = 27.74, *p* = 1.40 × 10^−5^; q = 1.75 × 10^−5^), and NPYR (H = 22.65, *p* = 1.97 × 10^−4^; q = 2.37 × 10^−4^) all remained significant after correction. By contrast, NDMA (H = 4.60, *p* = 0.2018; q = 0.2355) and NDEA (H = 3.69, *p* = 0.2931; q = 0.2931) did not differ across treatments, while NMOR showed only a borderline effect (H = 6.18, *p* = 0.1044; q = 0.1461). Regarding interaction effects, none of the analytes, including SUM, showed a significant processing × treatment interaction after FDR-BH correction, indicating that the impact of preservative formulation (notably Pr. + ASC + NO_2_) is consistent across processing conditions. Overall, these findings demonstrate that preservative formulation, especially the combination of ascorbate with nitrite, rather than processing or its interaction, is the main driver of nitrosamine variability in these experiments, highlighting a strong pro-oxidant role of ascorbate under nitrite-rich conditions.

To contextualize risk, we estimated the Margin of Exposure (MOE) by comparing daily exposure (from measured concentrations and portion sizes) with the group reference point BMDL_10_ = 10 µg/kg bw/day. Using 150 g for a 70 kg adult and 80 g for a 20 kg child, an average 5 µg/kg of total nitrosamines (based on digested meat and cooked + digested meat) gives MOE ≈ 930 (adult) and ≈500 (child). Since MOE ≥ 10,000 is generally considered of low concern, these values indicate a partial flag for concern and support mitigation measures, especially with frequent consumption or higher concentrations.

Mitigation strategies therefore become essential. Our results with spinach as a model vegetable provide a clear example: thanks to its high nitrate content and natural abundance of polyphenols, spinach modestly reduced nitrosamine levels during digestion, supporting the protective role of antioxidant-rich foods. Literature evidence aligns with this observation. Kuenzig et al. [32] first demonstrated that phenolic acids such as caffeic and ferulic acid can effectively block nitrosamine formation, while Honikel extensively reviewed the inhibitory action of ascorbate and erythorbate in curing processes. Wang et al. [33] showed that green tea polyphenols (GTP), grape seed extract (GSE), and α-tocopherol decreased residual nitrite, lipid oxidation, and NDMA levels in dry-cured bacon, while Zhou et al. [34] reported similar reductions in N-nitrosamines with rosemary, grape seed, and green tea extracts in smoked sausage. These studies, together with our results, underline the promise of combining meat products with antioxidant strategies as a way to mitigate nitrosamine exposure. Taken together, the data emphasize that although meat products alone can yield measurable nitrosamine levels under realistic processing and digestion conditions, dietary context profoundly shapes the final exposure. Pairing meat with antioxidant-rich vegetables appears to be a pragmatic approach to reduce nitrosamine risk, while ongoing research into natural extracts and innovative preservatives may provide technological tools to further limit formation. Integrating both nutritional choices and preservation strategies therefore offers the most effective pathway to minimize nitrosamine exposure from processed meats.

## 3. Materials and Methods

### 3.1. Chemical and Solvent

Acetonitrile (ACN; LC-MS, 99.9%), methanol (MeOH; LC-MS, 99.9%), dichloromethane (DCM; GC ≥ 99.9%), hexane (n-hexane; HPLC ≥ 97%), and hydrochloric acid (HCl ≥ 37%) were supplied by Honeywell (Muskegon, MI, USA). Formic acid (FA; LC-MS, 98%) was purchased from Merck (Darmstadt, Germany). For mass spectrometer calibration, ESI Negative Ion and ESI Positive Ion Pierce^®^ Calibration Solutions were used, provided by Thermo Scientific (Rockford, IL, USA). Deionized water was produced using the Arium^®^ Pro Lab Water System (Sartorius AG, Goettingen, Germany). Sodium chloride (NaCl ≥ 99.5%) was supplied by Honeywell (Muskegon, MI, USA). Pepsin from porcine gastric mucosa and gastric lipase from *Rhizopus oryzae*, Extrelut^®^ NT and Florisil^®^ Adsorbent for Chromatography and ammonium formate (≥99%) were provided by Merck.

### 3.2. Nitrosamine Standards

For this research, 15 different NAs were selected and considered because they have all been reported at least once in meat or meat-derived products in the literature, and analytical standards for these compounds were commercially available (either directly or upon specific requests to suppliers). This ensured both toxicological relevance and feasibility of robust analytical validation. The standards were supplied by Merck and Orion Scientific (Limena, Padova, Italy; Table 5). The standards were prepared by diluting the purchased stock solutions in methanol to a final concentration of approximately 0.25–0.5 mg/L. This mixed standard was further diluted to create calibration solutions ranging from 1.25–2.5 µg/L to 25–50 µg/L. The mixed stock standard was stored in the freezer and was stable for 2 months. The purchased solutions are stable for 8–12 months.

### 3.3. Sample Preparation

The meat was purchased directly from a local butcher (Salorno, South Tyrol, Italy), including: unprocessed meat (veal goulash, VS), processed and cured meat preserved with 15 mg/kg of NO_2_^−^ and 15 mg/kg of NO_3_^−^ (traditional pork *luganega* sausage, SN), processed meat preserved with a preparation containing 15 mg/kg of ascorbic acid (ASC; pork sausage, SA), processed meat preserved with a preparation containing ASC and further treated in the lab with 300 mg/kg of NO_2_^−^ (sausage treated with nitrites, SAN). Frozen spinach was purchased from a local supermarket (Salorno, South Tyrol, Italy) (average nitrate content 1–4 g/kg) [35]. Nitrites were added at a high dose (above permitted limits) in order to observe the protection provided by ascorbic acid in preventing formation in critical situations (equilibrium time 12 h a 4 °C).

The meat was processed in the following ways: raw meat (no treatment); digested raw meat (Section 3.3.1); grilled meat (Section 3.3.2); digested grilled meat (combination of Section 3.3.1 and Section 3.3.2); digested grilled meat combined, during digestion, with sautéed spinach (combination of Section 3.3.1 and Section 3.3.2, with the addition of cooked spinach).

#### 3.3.1. In Vitro Digestion Process (Stomach Simulation)

The in vitro gastric digestion protocol, adapted from Mandalari et al. [36], simulates human digestion and includes two phases: gastric and duodenal. For this study, only the gastric phase was applied, as NA formation predominantly occurs under acidic conditions. Lipid vesicles were excluded due to the low-fat content of the meat samples. Approximately 5 g of meat was weighed into 50 mL polypropylene tubes and mixed with 6.25 mL of NaCl solution (0.15 M, pH 2.5 adjusted with 1 M HCl) per gram of sample. The mixture was homogenized using an Ultra-Turrax^®^ (IKA-Werke GmbH & Co. KG, Staufen, Germany) and pH was re-adjusted to 2.5 to mimic gastric conditions. From the homogenate, 12.5 mL (equivalent to 2 g of meat) was taken, and the 12.5 µL of pepsin (from porcine gastric mucosa) and 125 µL of gastric lipase (from *Rhizopus oryzae*) enzymes were added. Samples were incubated at 37 °C for 2 h (minimum gastric emptying time) at 170 rpm, simulating gastric motility.

#### 3.3.2. Cooking Process

Meat samples were cut into small portions and grilled in a stainless-steel pan previously rinsed with Milli-Q (mQ) deionized water. Cooking time was approximately 2 min per side, with no oil added.

### 3.4. Extraction Method

The extraction protocol was adapted from Raoul et al. [15] with minor modifications. One gram of ground meat sample was homogenized using an Ultra-Turrax^®^ with 2 mL of 0.1 M NaOH for 1 min. The homogenate was then mixed with 2 g of Extrelut^®^ (Merck, Darmstadt, Germany) until fully absorbed. The resulting paste was transferred into a glass chromatography column (Merck) equipped with a porous frit at the bottom. Elution was performed using 7 mL of a hexane/DCM mixture (*v*/*v* 60:40). After elution, the dichloromethane (DCM) was evaporated at 51 °C for 4–6 h in a vacuum oven. The residual hexane phase was subjected to further purification through a Florisil^®^ SPE column (Merck), previously conditioned with 100% hexane. The sample was loaded onto the Florisil^®^ column, washed with 8 mL of 100% hexane, and allowed to dry. Subsequently, NAs were eluted with 6 mL of a DCM/hexane mixture (*v*/*v* 95:5). After elution, the DCM fraction was evaporated under a gentle nitrogen stream at 30 °C. The residue was reconstituted in 0.50 mL ACN/H_2_O (50:50, 0.1% FA), which was directly injected into the UHPLC-HRMS system (Thermo Fisher Scientific, Waltham, MA, USA) for analysis. All samples were extracted and analyzed in duplicate to ensure reproducibility.

### 3.5. LC-HRMS Analysis

#### 3.5.1. Method Validation

The instrumental limits in solvent (LOD) were estimated from the calibration regression according to the IUPAC/Eurachem approach, LOD = 3.3·σ/b and LOQ = 10·σ/b, where σ is the standard deviation of the residuals of the calibration and b the slope. Calibration curves were prepared at approximately 0.1, 0.5, 1, 5, 25 and 50 µg/L (slightly adjusted depending on analyte stock solutions). Recovery was assessed at a single spiking level of about 10 µg/kg in the meat matrix, performed in duplicate (N = 2). Recovery variability was reported as RSD%. Since matrix-matched calibration was not available, quantification was performed using solvent-based calibration. Results were corrected with recovery factors obtained from pre-extraction spiking experiments, which account for both extraction recovery and overall matrix effects, although the two contributions could not be distinguished separately. Uncertainty was estimated only for quantified nitrosamines in real samples, calculated as the relative standard deviation between replicates (u = SD/√2). To provide a representative value, the mean uncertainty across replicate groups was calculated for each analyte.

#### 3.5.2. Chromatographic Separation

The analysis was carried out using a Thermo UltiMate 3000 UHPLC (Thermo Fisher Scientific, Waltham, MA, USA) furnished with a Rheodyne 6-port automated switching valve and a pump module that allowed control of two independent fluid systems. The method used the same approach proposed by Nardin et al. [37]. The UHPLC was linked to a hybrid quadrupole orbitrap mass spectrometer (Q-Exactive) (Thermo Fisher Scientific, Waltham, MA, USA), equipped with a heated electrospray source (HESI-II) and an atmospheric pressure chemical ionization source (APCI). For chromatographic separation, an Acclaim Vanquish PA2 column (2.2 µm, 2.1 × 150 mm; Thermo Fisher Scientific) was used in conjunction with an online solid-phase extraction (SPE) column composed of divinylbenzene (DVB) (Dionex SolEx HRP, 2.1 × 20 mm, Thermo Fisher Scientific), which allowed for analyte pre-concentration and matrix cleanup. The eluents used for the online SPE column consisted of 0.1% formic acid in acetonitrile (A1) and water (B1). For the chromatographic column, the mobile phases included a 100 mM aqueous solution of formic acid with 20 mM ammonium formate (A2), acetonitrile (B2), and water (C2). The sample, 100 µL, was initially loaded onto the online SPE column using an isocratic flow composed of 5% A1 and 95% B1 at flow rate of 0.1 mL/min. Following loading, a 6-way valve directed the sample to waste during the initial flushing period. This condition was prolonged until 2.50 min. At 3.00 min, the valve switched to reverse-flush mode, connecting the SPE column to the analytical column, allowing the analytes to be eluted through the chromatographic gradient and carried to the detector. The gradient applied to the chromatographic column started with a flow rate of 0.3 mL/min, and an eluent composition of 10% A2, 5% B2, and 85% C2. At 12 min, the gradient shifted to 5% A2 and 95% B2, eliminating C2. This condition was held until 15 min, followed by a change to 1% A2 and 99% B2 at 15.1 min, which was maintained until 17.5 min. Subsequently, the initial condition was re-established at 17.60 min (10% A2, 5% B2, 85% C2) and kept stable until 21 min to ensure full re-equilibration of the column. At 18.1 min the 6-way valve switched and directed the flow to waste for reconditioning the SPE column.

Injections were performed with a volume of 5 µL. The autosampler was maintained at 5 °C, while the column compartment temperature was set to 40 °C throughout the analysis.

#### 3.5.3. HESI (Heated-Electrospray Ionization) Source Settings

The mass spectrometer was configured with a HESI source. HESI is a widely used variant of standard electrospray ionization (ESI), in which additional heating improves droplet desolvation and enhances ionization efficiency, particularly for polar and thermolabile compounds. The spray voltage was set to 4.5 kV, while the capillary temperature was maintained at 200 °C. The sheath gas flow rate was adjusted to 60 arbitrary units, and the auxiliary gas flow rate was set to 10 arbitrary units. Additionally, a sweep gas flow of 1 was used. The maximum spray current was 2 µA, and the probe heater temperature was regulated at 300 °C. The S-Lens RF level was set to 55.

#### 3.5.4. APCI (Atmospheric Pressure Chemical Ionization) Source Settings

For the APCI ion source configuration, the capillary temperature was kept at 200 °C, the sheath gas flow rate was adjusted to 45 arbitrary units, and the auxiliary gas flow rate was 10 arbitrary units. A sweep gas flow of 1 was also applied. The corona current was set to 2 µA, with the probe heater temperature at 300 °C. The S-Lens RF level was maintained at 55, the same as for the HESI source.

#### 3.5.5. Detection Conditions

Mass spectra were acquired using a Full Mass–Data Dependent MS^2^ analysis in both positive (ESI+) and negative (ESI-) ionization modes, with polarity switching enabled during the run. For the Full MS Scan, the resolution was set to 70,000 full width at half-maximum (FWHM, calculated for *m*/*z* 200, 1.5 Hz), with an AGC (Automatic Gain Control) target of 3e6, a maximum injection time (IT) of 100 ms, and a scan range of 50–300 m/z for the negative mode and 50–500 m/z for the positive mode. For the Data Dependent MS^2^ Scan, the resolution was set to 17,500 FWHM, with an AGC target of 1e5, a maximum IT of 50 ms, and a normalized collision energy (NCE) of 40 eV. The identification of the analytes was based on their exact mass (with a mass accuracy error of less than 5 ppm), isotopic pattern, and fragmentation evaluation. The mass spectrometer was calibrated daily (Pierce ESI pos/neg).

## 4. Conclusions

In summary, this study highlights both the analytical and biochemical complexity that is associated with nitrosamine detection in meat matrices. Because different nitrosamines possess different physicochemical characteristics, the use of a single ionization method can lead to biased or partial profiling. Therefore, the concurrent use of HESI (for NDPhA, NPIP and NMOR, all VNAs) and APCI (for other VNAs and NVNAs) is strongly recommended for improved analytical coverage and sensitivity. This study demonstrates that nitrosamine formation in meat products is a multifactorial process that is reliant on the nature of the present additives, the processing conditions (e.g., cooking), and the digestive environment. Particular attention should be paid to the role of ascorbate: traditionally regarded as an inhibitor of nitrosamine formation, our results confirm a dual, dose-dependent function. At low or absent nitrite levels, ascorbate acts as an effective inhibitor, whereas in the presence of high nitrite concentrations (150 mg/kg, i.e., above regulatory limits), it exerts a pro-oxidant effect, likely through the catalytic generation of nitrosating intermediates. It is important to note that ascorbate cannot inhibit the growth of *Clostridium botulinum*, and therefore, the combination with nitrites will always be necessary. For future research, it would be interesting to investigate possible plant extracts such as rosemary or green tea, which in any case should be combined with nitrites, or to explore novel options such as bacteriocins.

Spinach was selected as a model vegetable because of its naturally high nitrate content and its dietary relevance but also because of the combination with its polyphenol content, which could reflect the use of natural extracts in combination with nitrates. Although its inclusion during digestion modestly reduced nitrosamine concentrations, it nevertheless introduced additional reactive complexity, suggesting that other nitrate-rich vegetables may also influence nitrosamine formation and should be considered in future studies. This confirms that the approach of using natural extracts could be of interest, especially when applied with purified extracts, but at the same time, it highlights that the potential and infinite food combinations should be carefully considered, as they will inevitably modify nitrosamine production during digestion. Moreover, we evaluated that cooking is not sufficient to cause extensive nitrosation but enhances it upon subsequent gastric digestion. Variabilities between replicates indicate that formation is not necessarily systematic but can be substantial. Furthermore, the selective presence or absence of particular nitrosamines among groups can be explained by their thermal capability or relative stability under acidic or thermal conditions. Among them, NDMA consistently emerges as the most relevant marker of dietary nitrosamine exposure, in agreement with previous literature and with EFSA’s identification of NDMA as the reference compound for risk assessment. These findings warrant the need to reassess, critically, the use of nitrites in processed food but also to carefully examine the effects of alternative preservatives before considering them totally safe. Future research should focus on optimizing extraction methods to improve the recovery rates of more polar nitrosamines. Finally, the UHPLC-HRMS method could be applied in regulatory or industrial contexts for further exploring the analytical profile of nitrosamines, which has emerged from our work as highly diverse and still largely unexplored.

## Figures and Tables

**Table 1:** Retention comparison between Divinylbenzene (DVB) and Polystyrene Divinylbenzene (PEP; PS-DVB with urea groups-mixed-mode) SPE column.

Compound	Retention Time (min)	
	DVB	PEP
4-(N-nitrosomethylamino)-1-(3-pyridyl)-1-butanone	0.39	0.41
N-nitrosomorpholine	0.39	0.4
N-nitrosonornicotine	0.4	0.41
N-nitrosodimethylamine	0.4	0.41
N-nitrosopyrrolidine	0.4	0.4
N-nitroso-N-methylethylamine	0.43	0.43
N-nitrosodiethylamine	0.45	0.45
N-nitrosopiperidine	0.47	0.46
N-nitrosodi-n-propylamine	0.54	0.55
N-nitrosodi-n-butylamine	0.66	0.62
3-nitrosothiazolidine-4-carboxylic acid	1.34	1.76
N-nitrosodiphenylamine	2.02	0.73
N-nitroso-L-proline	2.14	0.75

**Table 2:** Comparison of chromatographic parameters across tested columns.

	Retention Time (min)				K′				Asymmetry (EP)				Plates (EP)			
	Poroshell 120	APP	Hypersil G	PA2	Poroshell 120	APP	Hypersil G	PA2	Poroshell 120	APP	Hypersil G	PA2	Poroshell 120	APP	Hypersil G	PA2
4-(N-nitrosomethylamino)-1-(3-pyridyl)-1-butanone	6.6	10.7	8.4	10.2	64.8	106.3	83.3	101	1.0	0.7	n.a.	0.7	440	2133	2388	2530
N-nitrosonornicotine	1.6	3.2	2.3	2.4	14.5	31.4	21.5	23.4	0.9	1.0	1.1	0.7	99	259	153	227
N-nitrososarcosine	16.4	19.5	18.8	1.8	163	194	187	16.9	0.9	0.7	0.7	0.7	11,803	39,086	6135	556
N-nitroso-L-proline	16.6	19.5	n.a.	2.0	165	194	n.a.	19.3	1.0	1.0	n.a.	1.0	34,235	319,561	n.a.	6602
N-nitrosodimethylamine	1.3	3.1	1.9	2.5	11.8	30.5	17.6	23.7	2.1	1.4	1.5	1.3	93	506	155	307
3-nitrosothiazolidine-4-carboxylic acid	19.2	0.5	23.4	2.2	191	4	233	20.9	0.6	1.4	0.8	2.5	11,523	51	99,594	1275
N-nitrosomorpholine	1.6	3.8	2.3	3.1	14.9	36.8	22.2	29.8	1.9	1.3	1.2	1.4	136	257	80	176
N-nitroso-2-methylthiazolidine-4-carboxylic acid	1.1	2.9	1.6	2.1	10.2	27.5	15.1	20.5	1.2	0.8	1.2	0.8	110	1111	451	569
N-nitroso-N-methylethylamine	2.0	4.5	2.8	3.7	18.8	44.2	27.5	35.6	1.9	1.1	1.0	1.2	159	345	125	251
N-nitrosopyrrolidine	16.6	4.4	2.9	3.7	165	43.1	27.6	35.7	0.6	1.8	1.6	2.4	1864	285	108	215
N-nitrosodiethylamine	4.1	8.5	5.7	6.9	39.8	83.8	55.9	67.9	1.5	0.9	0.9	0.9	456	637	234	469
N-nitrosopiperidine	5.2	10.5	7.1	8.6	50.7	104	69.8	85.0	2.2	1.2	1.1	1.3	420	854	348	600
N-nitrosodi-n-propylamine	10.4	14.1	12.6	13.4	103	140	125	133	1.3	1.7	1.7	1.7	8806	21,756	15,996	20,242
N-nitrosodi-n-butylamine	11.5	15.2	13.7	14.5	114	151	136	144	1.4	1.5	1.7	1.6	11,631	36,539	27,226	33,737
N-nitrosodiphenylamine	11.6	15.4	13.9	14.7	115	153	138	146	1.2	1.3	1.4	1.3	13,317	45,797	32,130	41,320

Note: APP = Accucore Polar Premium; n.a.= not applicable.

**Table 3:** Validation parameters for HESI and APCIs.

Compound	RT (min)	[M+H]^+^ (*m*/*z*)	Fragments (*m*/*z*)	LOD (µg/kg)		R^2^ (LOD-50 µg/L)		Recovery (%; 10 µg/L)		RSD (%; N = 2)	
				APCI	HESI	APCI	HESI	APCI	HESI	APCI	HESI
N-nitrosodimethylamine	4.3	75.0553	n.d.	1.0	1.5	0.998	0.989	37	n.d.	9	n.d.
N-nitrososarcosine	4.7	119.0451	92.050	1.0	25	0.999	0.999	45	40	9	10
N-nitrosonornicotine	4.9	178.0975	120.068	2.0	0.5	0.999	0.996	87	62	13	14
N-nitrosomorpholine	5.3	117.0659	87.068	2.0	0.2	0.998	0.994	10	75	19	5
N-nitroso-L-proline	5.3	145.0608	121.966	0.5	5.0	0.998	0.996	31	n.d.	6	n.d.
N-nitroso-N-methylethylamine	5.5	89.0709	61.040	0.1	2.0	0.992	0.990	48	50	2	2
N-nitrosopyrrolidine	5.5	101.0709	55.055	0.1	0.3	0.996	0.999	36	49	10	13
N-nitrosodiethylamine	7.4	103.0866	75.056	0.1	5.0	0.999	0.999	31	13	4	13
4-(N-nitrosomethylamino)-1-(3-pyridyl)-1-butanone	7.5	208.1081	122.060	3.0	0.5	0.985	0.989	85	15	8	12
N-nitrosopiperidine	8.0	115.0866	69.070	0.2	2.0	0.989	0.999	27	37	15	7
N-nitrosodi-n-propylamine	10.6	131.1179	89.071	0.5	1.0	0.998	0.999	37	26	10	11
N-nitrosodi-n-butylamine	12.7	159.1492	103.087	1.0	3.0	0.997	0.997	35	9	1	10
N-nitrosodiphenylamine	13.4	199.0866	169.088	n.d.	0.5	n.d.	0.995	n.d.	35	n.d.	4
3-nitrosothiazolidine-4-carboxylic acid	6.6	161.0026	71.025	1.0	5.0	0.999	0.999	31	38	3	3
N-nitroso-2-methylthiazolidine-4-carboxylic acid	8.0	175.0183	71.025	1.0	5.0	0.997	0.995	155	25	4	10

Note: RT, Retention Time; LOD, Limit of Detection; RSD, Relative Standard Deviation; APCI, Atmospheric Pressure Chemical Ionization; HESI, Heated Electrospray Ionization; n.d. = not detected.

**Table 4:** NA presence across different meat preparations and treatments. The color gradient from orange to red indicates increasing concentration.

		N-Nitrosodiethylamine	N-Nitrosodimethylamine	N-Nitrosodi-n-Butylamine	N-Nitrosodi-n-Propylamine	N-Nitrosopiperidine	N-Nitrosopyrrolidine	N-Nitrosomorpholine	SUM
**RAW MEAT**	Not Treated			3.0					3.0
	Not Treated			2.0					2.0
	Pr. + ASC			2.0				2.8	4.8
	Pr. + ASC			2.0				3.9	5.9
	Pr. + NO_2_ + NO_3_		6.9						6.9
	Pr. + NO_2_ + NO_3_		5.0						5.0
	Pr. + ASC + NO_2_			6.0	1.2				7.2
	Pr. + ASC + NO_2_		23	32	6.4				62
**DIGESTED RAW MEAT**	Not Treated								
	Not Treated								
	Pr. + ASC	0.6							0.6
	Pr. + ASC	1.3							1.3
	Pr. + NO_2_ + NO_3_	1.0	8.1	4.0					13
	Pr. + NO_2_ + NO_3_		4.1	2.0					6.1
	Pr. + ASC + NO_2_	1.3		14	1.7	31	0.3		48
	Pr. + ASC + NO_2_	0.6		12	1.7	55	1.4		70
**COOKED**	Not Treated								
	Not Treated								
	Pr. + ASC								
	Pr. + ASC								
	Pr. + NO_2_ + NO_3_			2.0	1.2				3.2
	Pr. + NO_2_ + NO_3_			1.0	0.5				1.5
	Pr. + ASC + NO_2_		4.1	12	4.1	10	0.8		31
	Pr. + ASC + NO_2_			18		14	1.4		33
**COOKED AND DIGESTED**	Not Treated								
	Not Treated								
	Pr. + ASC	1.0			0.6				1.5
	Pr. + ASC			2.0					2.0
	Pr. + NO_2_ + NO_3_								
	Pr. + NO_2_ + NO_3_								
	Pr. + ASC + NO_2_	0.6		7.0	2.9	56	9.6		76
	Pr. + ASC + NO_2_	0.7	2.9	5.0	2.3	33	1.7		46
**COOKED AND DIGESTED WITH SPINACH**	Not Treated	1.0							1.0
	Not Treated	1.1							1.0
	Pr. + ASC		8.7						8.7
	Pr. + ASC		6.4						6.4
	Pr. + NO_2_ + NO_3_	0.6							0.6
	Pr. + NO_2_ + NO_3_								
	Pr. + ASC + NO_2_	1.0		4.0	8.1	4.4			17
	Pr. + ASC + NO_2_	0.6		2.0	2.3	2.2			7.2

**Table 5:** Nitrosamine standards.

Compound	Molecular Formula	IARC Classification	Supplier *
*Volatile Nitrosamines*			
N-nitrosodimethylamine (NDMA)	C_2_H_6_N_2_O	2A	a
N-nitroso-N-methylethylamine (NMEA)	C_3_H_8_N_2_O	2B	a
N-nitrosopyrrolidine (NPYR)	C_4_H_8_N_2_O	2B	a
N-nitrosopiperidine (NPIP)	C_5_H_10_N_2_O	2B	a
N-nitrosomorpholine (NMOR)	C_4_H_8_N_2_O_2_	2B	a
N-nitrosodi-n-propylamine (NDPA)	C_6_H_14_N_2_O	2B	a
N-nitrosodi-n-butylamine (NDBA)	C_8_H_18_N_2_O	2B	a
N-nitrosodiphenylamine (NDPhA)	C_12_H_10_N_2_O	3	a
N-nitrosodiethylamine (NDEA)	C_4_H_10_N_2_O	2A	a
N-nitrosonornicotine (NNN)	C_9_H_11_N_3_O	1	a
4-(N-nitrosomethylamino)-1-(3-pyridyl) -1-butanone (NNK)	C_10_H_13_N_3_O_2_	1	a
*Non-Volatile Nitrosamines*			
N-nitrososarcosine (NSAR)	C_3_H_6_N_2_O_3_	2B	b
N-nitroso-L-proline (NPRO)	C_5_H_8_N_2_O_3_	3	b
N-nitrosothiazolidine-4-carboxylic acid (NTCA)	C_4_H_6_N_2_O_3_S	-	b
N-nitroso-2-methylthiazolidine 4-carboxylic acid (NMTCA)	C_5_H_8_N_2_O_3_S	-	b

* a = Sigma Aldrich − Merck; b = ORION Scientific srl; 1 = carcinogenic; 2A = probably carcinogenic to humans; 2B = possibly carcinogenic; 3 = unclassifiable.

## Data Availability

Data is contained within the article. Further inquiries can be directed to the corresponding author.

## Abbreviations

The following abbreviations are used in this manuscript:

NA	Nitrosamine
FWHM	full width at half-maximum
HRMS	High-Resolution Mass Spectrometry
NDBA	N-nitrosodi-n-butylamine
NDEA	N-nitrosodiethylamine
NDMA	N-nitrosodimethylamine
NDPA	N-nitrosodi-n-propylamine
NDPhA	N-nitrosodiphenylamine
NNK	4-(N-nitrosomethylamino)-1-(3-pyridyl)-1-butanone
NMEA	N-nitroso-N-methylethylamine
NMOR	N-nitrosomorpholine
NMTCA	N-nitroso-2-methylthiazolidine-4-carboxylic acid
NNN	N-nitrosonornicotine
NPIP	N-nitrosopiperidine
NPRO	N-nitroso-L-proline
NPYR	N-nitrosopyrrolidine
NSAR	N-nitrososarcosine
NTCA	3-nitrosothiazolidine-4-carboxylic acid
NVNA	non-volatile nitrosamine
UHPLC	Ultra-High Performance Liquid Chromatography
VNA	Volatile nitrosamine
